# Supplementary figures and images for: Deep learning analysis of fMRI data for predicting Alzheimer’s Disease: A focus on convolutional neural networks and model interpretability
Source: PLoS One. 2024 Dec 4;19(12):e0312848. doi: 10.1371/journal.pone.0312848 (PMC11616848; doi:10.1371/journal.pone.0312848)

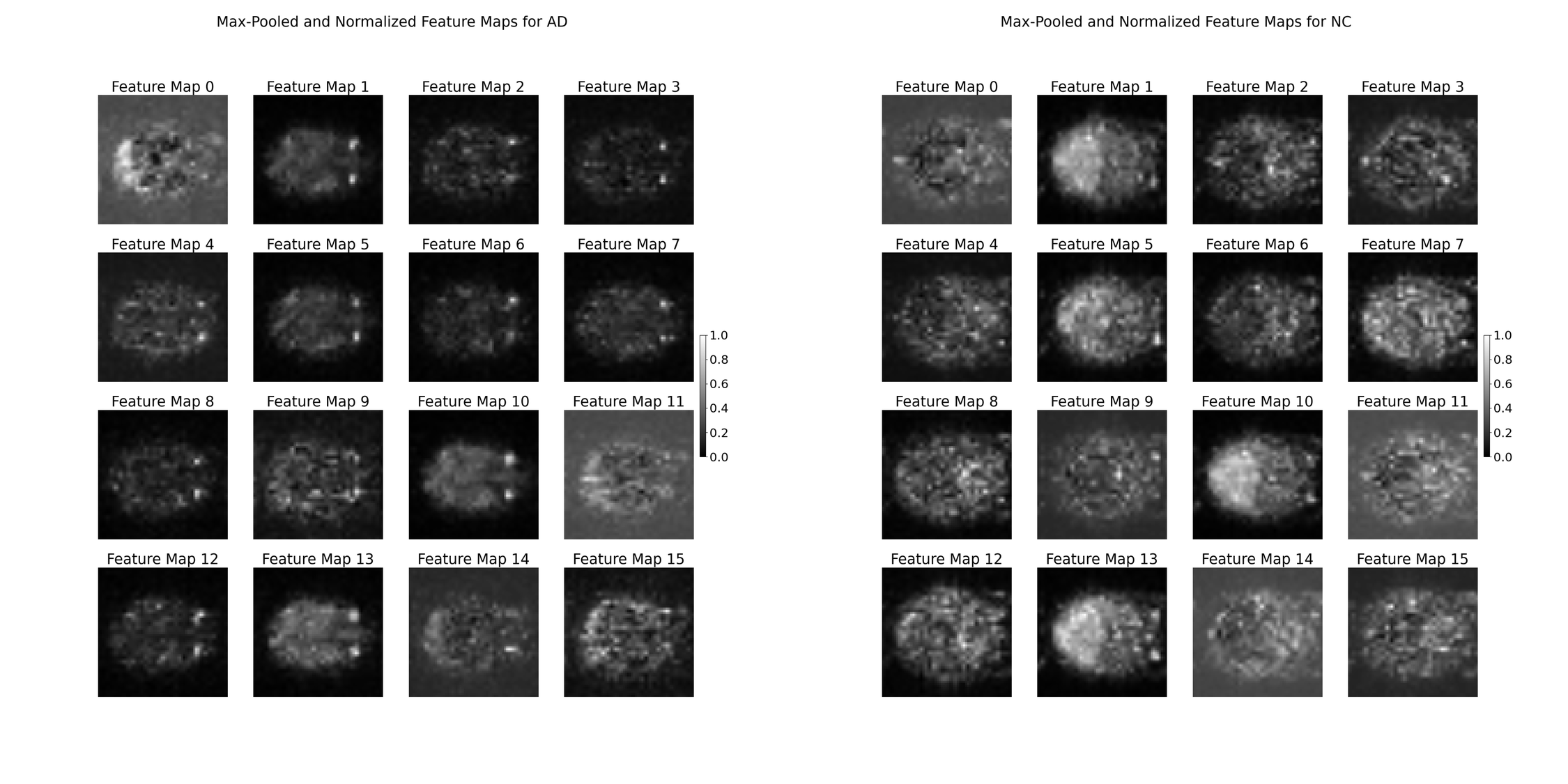

Supplement: S1 Fig — The left panel displays feature maps from a participant diagnosed with AD. The right panel shows feature maps from a cognitively normal (NC) participant. S1 of S3 Figs. (TIF) [file pone.0312848.s001.tif]

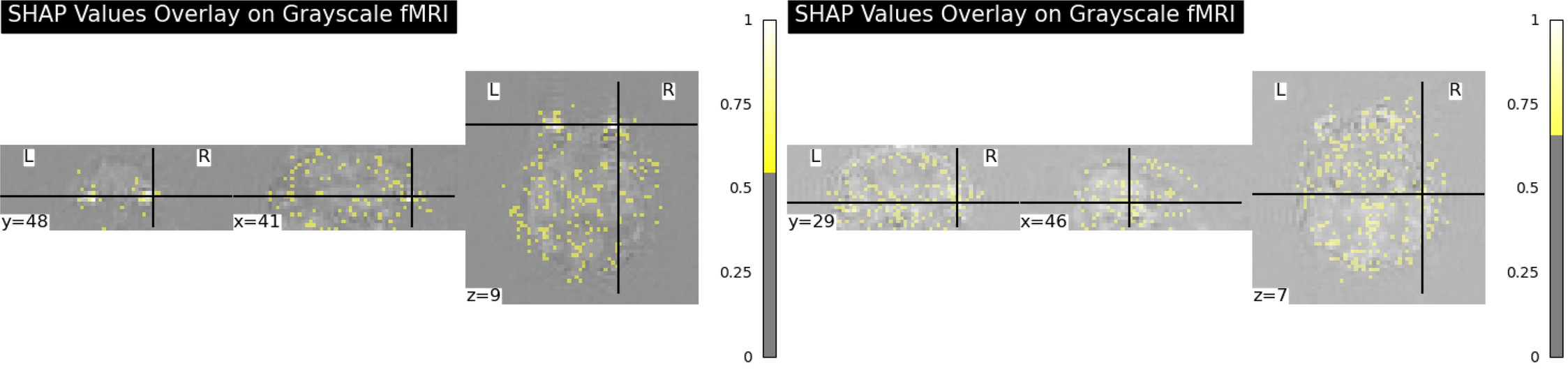

Supplement: S2 Fig — (1/3) Each pair of images shows the spatial distribution of influential voxels: the left image for a participant diagnosed with AD and the right image for a cognitively normal (NC) participant. Through these comparative visualizations, potential AD-impacted regions and the model’s diagnostic accuracy are highlighted. (TIF) [file pone.0312848.s002.tif]

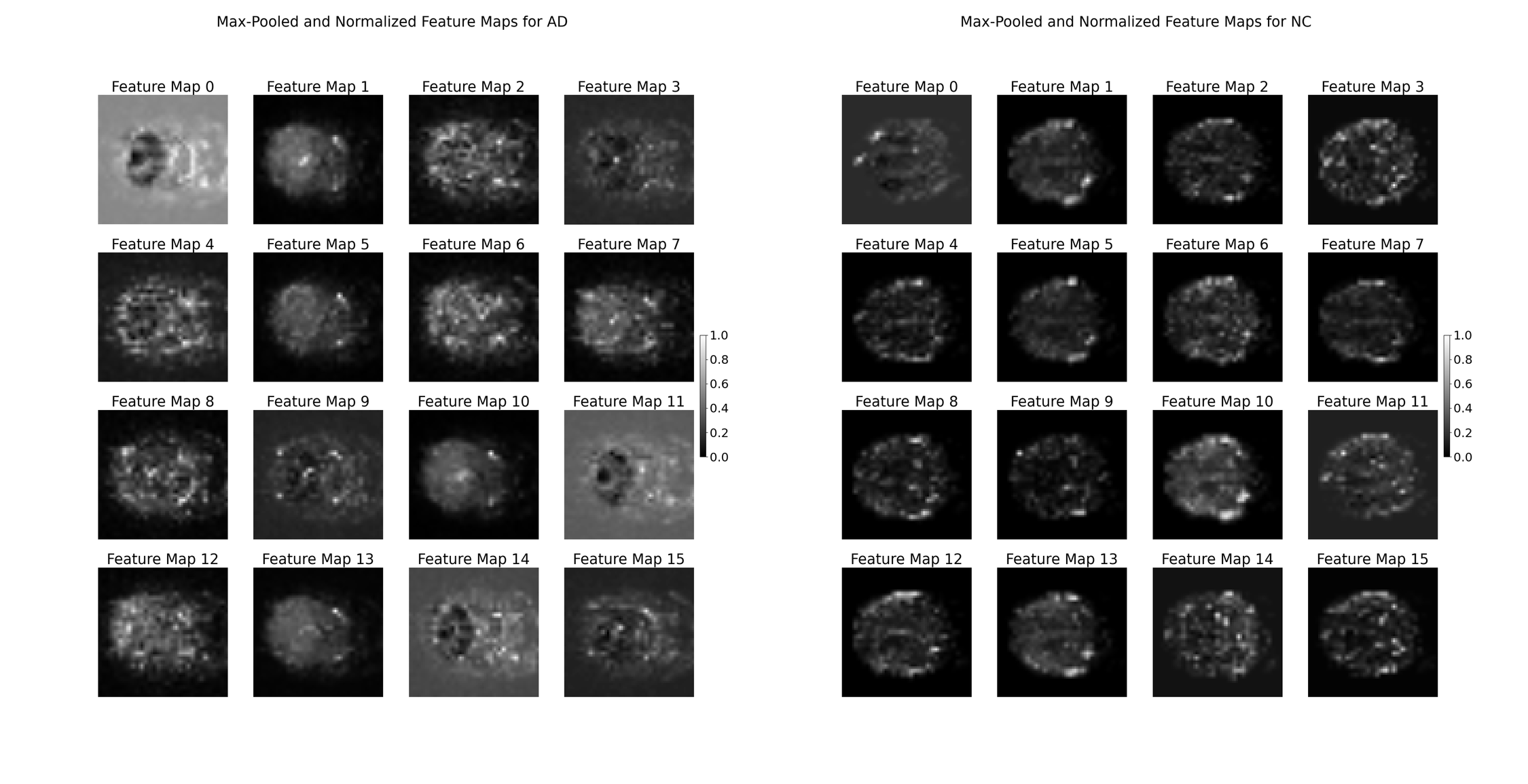

Supplement: S3 Fig — The left panel displays feature maps from a participant diagnosed with AD. The right panel shows feature maps from a cognitively normal (NC) participant. (TIF) [file pone.0312848.s003.tif]

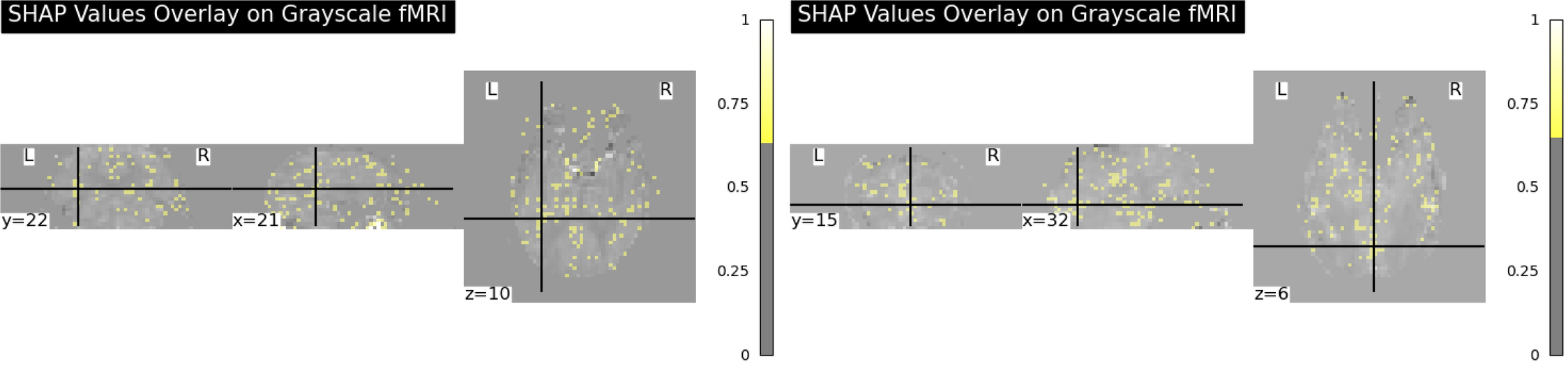

Supplement: S4 Fig — Each pair of images shows the spatial distribution of influential voxels: the left image for a participant diagnosed with AD and the right image for a cognitively normal (NC) participant. Through these comparative visualizations, potential AD-impacted regions and the model’s diagnostic accuracy are highlighted. (TIF) [file pone.0312848.s004.tif]

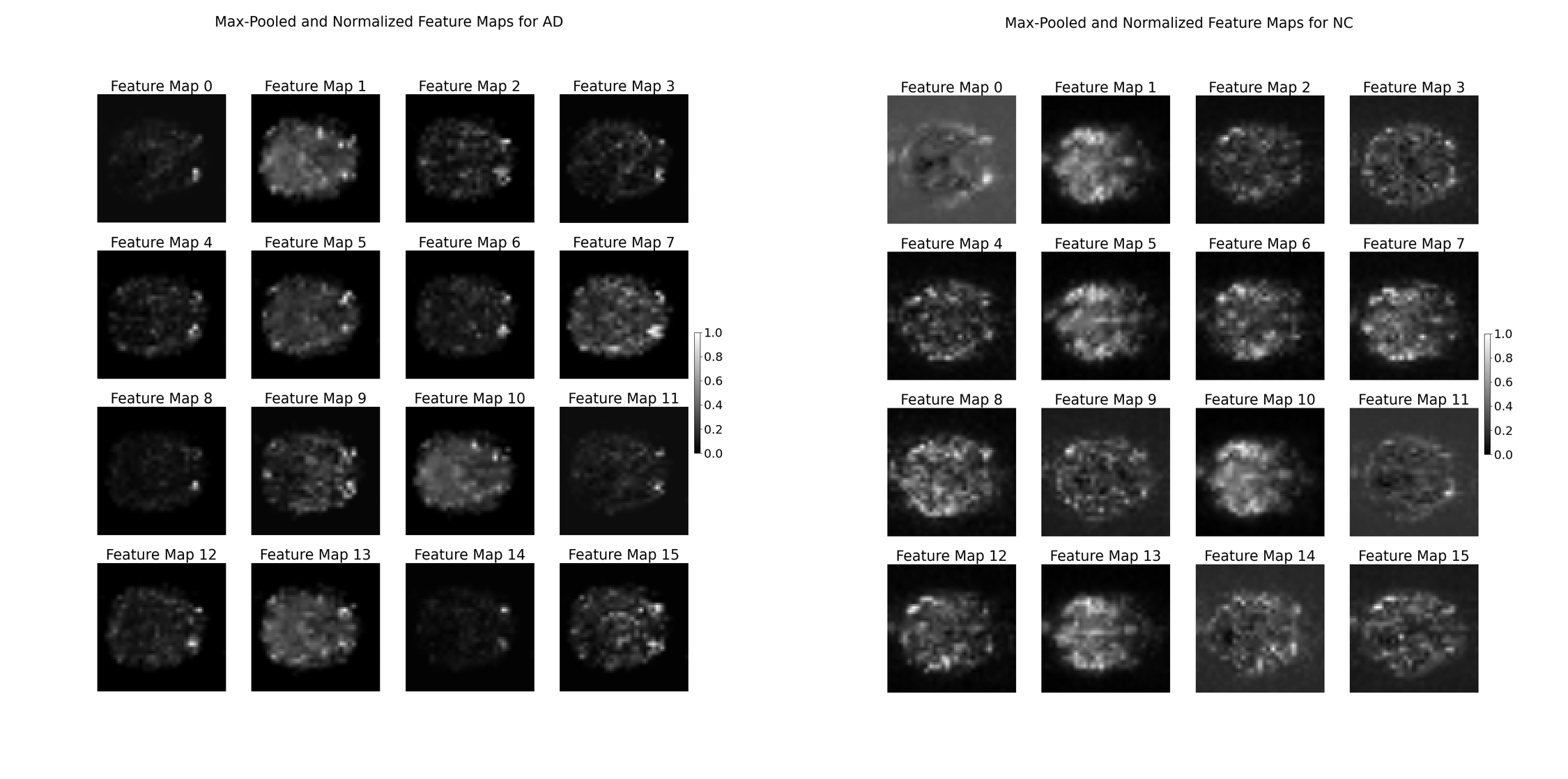

Supplement: S5 Fig — The left panel displays feature maps from a participant diagnosed with AD. The right panel shows feature maps from a cognitively normal (NC) participant. (TIF) [file pone.0312848.s005.tif]

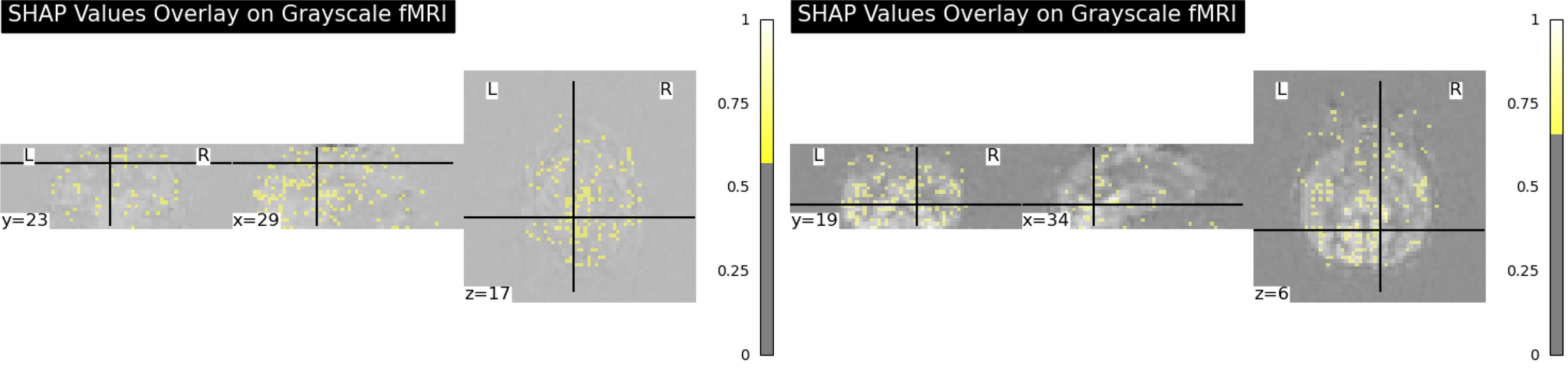

Supplement: S6 Fig — Each pair of images shows the spatial distribution of influential voxels: the left image for a participant diagnosed with AD and the right image for a cognitively normal (NC) participant. Through these comparative visualizations, potential AD-impacted regions and the model’s diagnostic accuracy are highlighted. (TIF) [file pone.0312848.s006.tif]

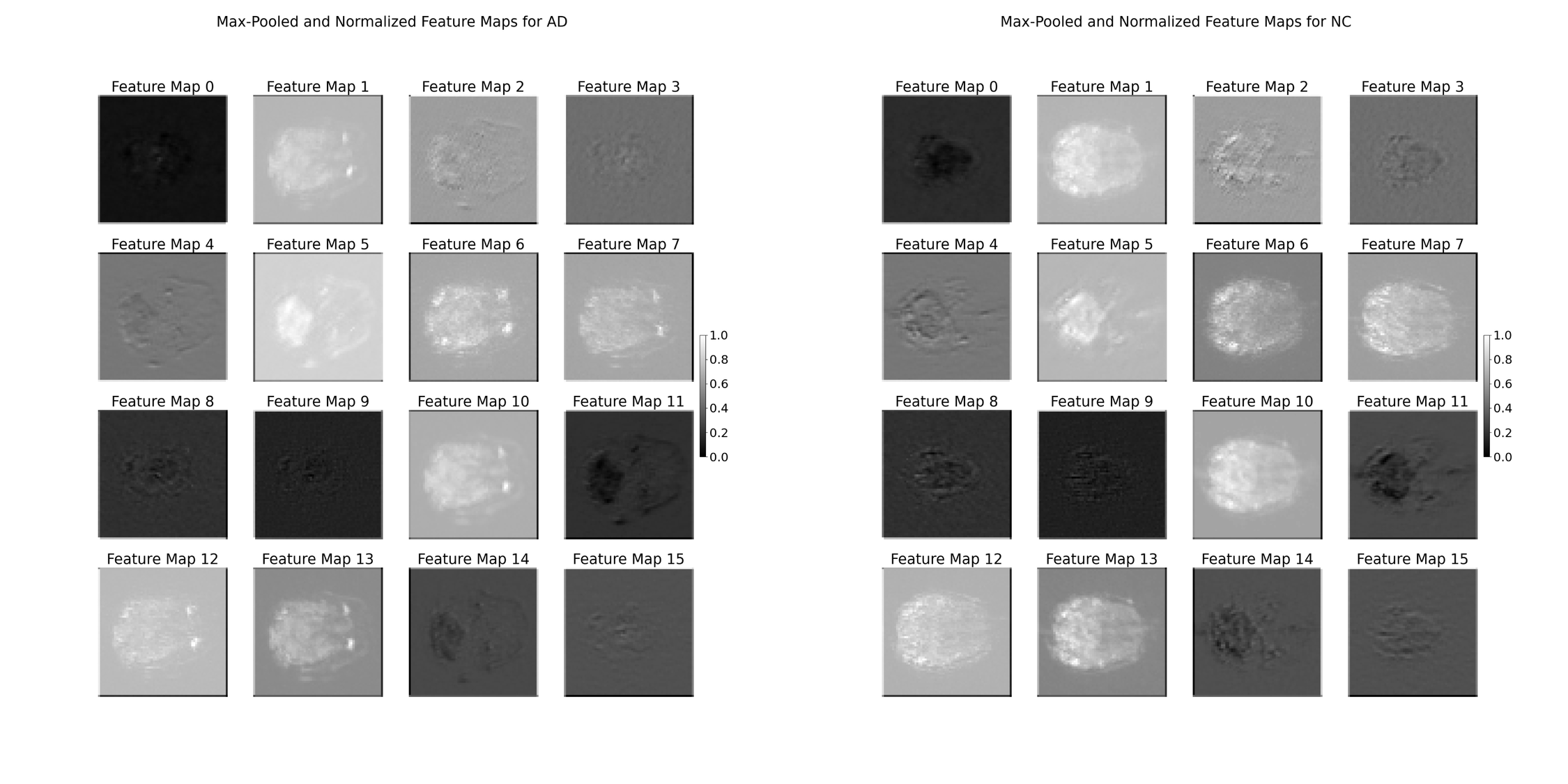

Supplement: S7 Fig — The left panel displays feature maps from a participant diagnosed with AD. The right panel shows feature maps from a cognitively normal (NC) participant. (TIF) [file pone.0312848.s007.tif]

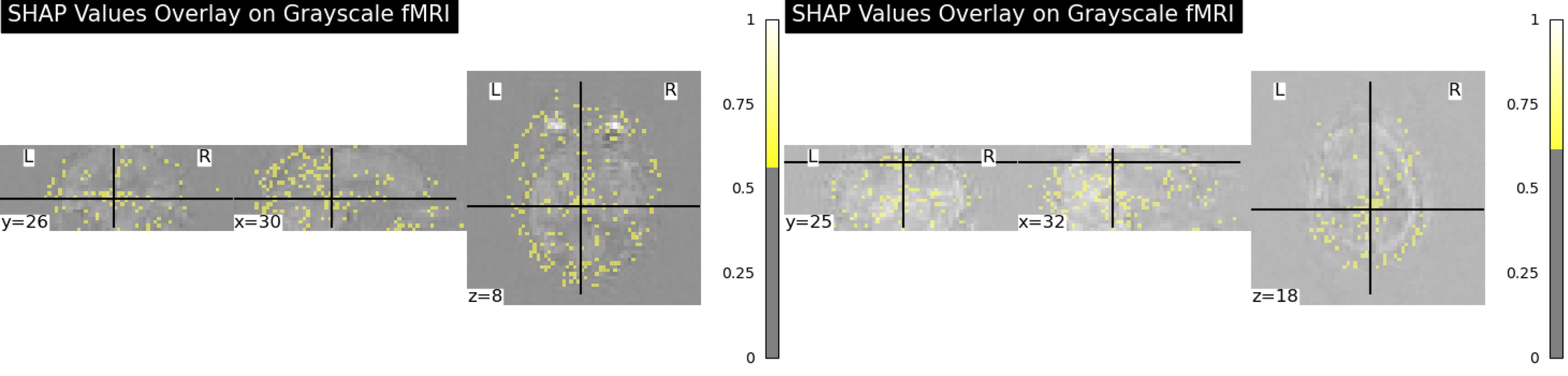

Supplement: S8 Fig — Each pair of images demonstrates the spatial distribution of influential voxels: the left image for a participant diagnosed with AD and the right image for a cognitively normal (NC) participant. (TIF) [file pone.0312848.s008.tif]

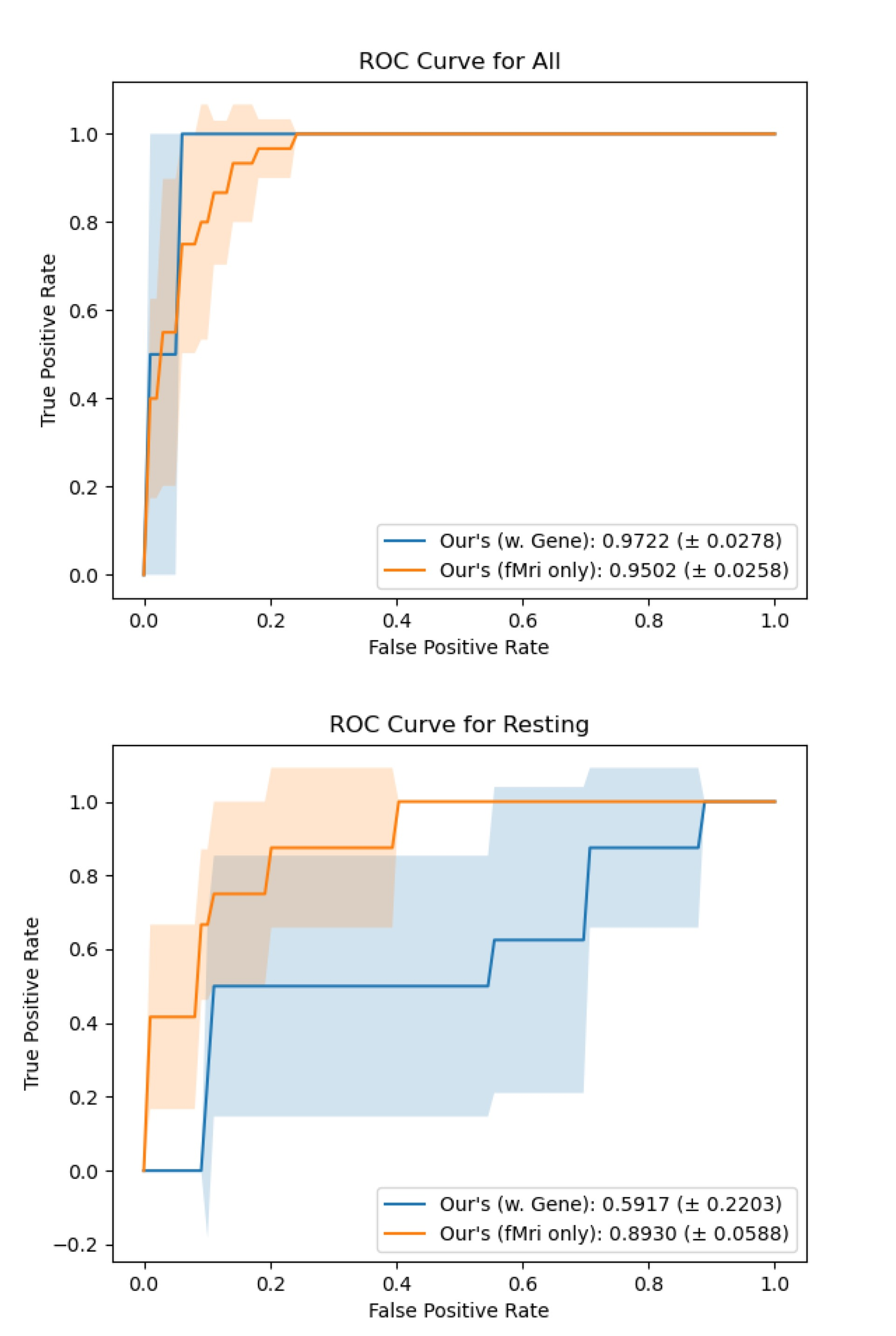

Supplement: S9 Fig — Receiver Operating Characteristic (ROC) curves comparing the performance of models with different input data, specifically our CNN with and without gene data as additional inputs across different datasets. Each curve represents the trade-off between the True Positive Rate (TPR) and False Positive Rate (FPR) for a specific model. (TIF) [file pone.0312848.s009.tif]

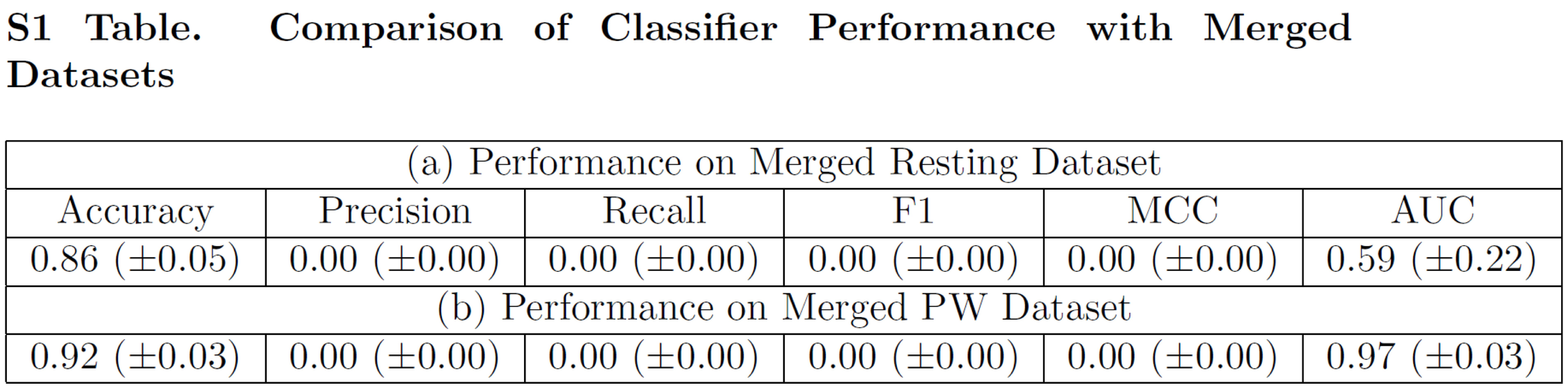

Supplement: S1 Table — This table presents a comparative analysis focusing on the performance of our model with merged input data from Resting and PW datasets. The performance metrics include Accuracy, Precision, Recall, F1, Matthews Correlation Coefficient (MCC), and Area Under the Curve (AUC). The results showcase the models performance does not significantly improved with gene as additional input. (TIF) [file pone.0312848.s010.tif]
