# Supplementary material for: Deep learning analysis of fMRI data for predicting Alzheimer’s Disease: A focus on convolutional neural networks and model interpretability
Source: PLoS One. 2024 Dec 4;19(12):e0312848. doi: 10.1371/journal.pone.0312848 (PMC11616848; doi:10.1371/journal.pone.0312848)
Supplement: Attachment — Submitted filename: Response to editorial and reviewer comments.docx [file pone.0312848.s011.docx]

# Response to editorial and reviewer comments

We sincerely thank the editorial team and the reviewers for providing constructive feedback on our manuscript. In the following section, we will provide responses to the editorial and reviewer comments.

### Editors' comments:

**When submitting your revision, we need you to address these additional requirements.**

**1. Please ensure that your manuscript meets PLOS ONE's style requirements, including those for file naming. The PLOS ONE style templates can be found at**
[**https://journals.plos.org/plosone/s/file?id=wjVg/PLOSOne_formatting_sample_main_body.pdf**](https://nam12.safelinks.protection.outlook.com/?url=https%3A%2F%2Fjournals.plos.org%2Fplosone%2Fs%2Ffile%3Fid%3DwjVg%2FPLOSOne_formatting_sample_main_body.pdf&data=05%7C02%7Cxiao.zhou%40yale.edu%7C600aa7c79f8e4801e9f708dcad9ab9f4%7Cdd8cbebb21394df8b4114e3e87abeb5c%7C0%7C0%7C638576127264858514%7CUnknown%7CTWFpbGZsb3d8eyJWIjoiMC4wLjAwMDAiLCJQIjoiV2luMzIiLCJBTiI6Ik1haWwiLCJXVCI6Mn0%3D%7C0%7C%7C%7C&sdata=%2BrurcxlenM4RIIuaIfKyhBBNeeiwChAi6EJrYOGjJxw%3D&reserved=0) **and**
[**https://journals.plos.org/plosone/s/file?id=ba62/PLOSOne_formatting_sample_title_authors_affiliations.pdf**](https://nam12.safelinks.protection.outlook.com/?url=https%3A%2F%2Fjournals.plos.org%2Fplosone%2Fs%2Ffile%3Fid%3Dba62%2FPLOSOne_formatting_sample_title_authors_affiliations.pdf&data=05%7C02%7Cxiao.zhou%40yale.edu%7C600aa7c79f8e4801e9f708dcad9ab9f4%7Cdd8cbebb21394df8b4114e3e87abeb5c%7C0%7C0%7C638576127264864622%7CUnknown%7CTWFpbGZsb3d8eyJWIjoiMC4wLjAwMDAiLCJQIjoiV2luMzIiLCJBTiI6Ik1haWwiLCJXVCI6Mn0%3D%7C0%7C%7C%7C&sdata=BfzZnDtmYBNTlD27T97799NFKN2DLPYAm4wxnIjevGE%3D&reserved=0)

Thank you for providing the style templates, which have been instrumental in refining our manuscript to meet the journal's standards. We have reviewed the templates and guidelines in the links and have made additional changes (i.e. figure 1 -> Fig. 1, section relocation, etc. ) to ensure that our manuscript adheres to these requirements. We have also updated our file naming to ensure they meet the journal’s requirements.

Additionally, the current format of our manuscript was built based on the LaTeX template provided by the journal at https://journals.plos.org/plosone/s/latex. Please let us know if this format is outdated or not applicable. We hope the manuscript now fully complies with PLOS ONE's formatting requirements but are prepared to make any further adjustments if needed.

**2. For studies involving third-party data, we encourage authors to share any data specific to their analyses that they can legally distribute. PLOS recognizes, however, that authors may be using third-party data they do not have the rights to share. When third-party data cannot be publicly shared, authors must provide all information necessary for interested researchers to apply to gain access to the data. (**[**https://journals.plos.org/plosone/s/data-availability#loc-acceptable-data-access-restrictions**](https://nam12.safelinks.protection.outlook.com/?url=https%3A%2F%2Fjournals.plos.org%2Fplosone%2Fs%2Fdata-availability%23loc-acceptable-data-access-restrictions&data=05%7C02%7Cxiao.zhou%40yale.edu%7C600aa7c79f8e4801e9f708dcad9ab9f4%7Cdd8cbebb21394df8b4114e3e87abeb5c%7C0%7C0%7C638576127264870714%7CUnknown%7CTWFpbGZsb3d8eyJWIjoiMC4wLjAwMDAiLCJQIjoiV2luMzIiLCJBTiI6Ik1haWwiLCJXVCI6Mn0%3D%7C0%7C%7C%7C&sdata=vUT1AsnZE6oqWd9%2FnxIVom5Y1DinHhQmLhVmzYSJJWE%3D&reserved=0)**)**

**For any third-party data that the authors cannot legally distribute, they should include the following information in their Data Availability Statement upon submission:
1) A description of the data set and the third-party source
2) If applicable, verification of permission to use the data set
3) Confirmation of whether the authors received any special privileges in accessing the data that other researchers would not have
4) All necessary contact information others would need to apply to gain access to the data**

Thank you for clarification on the requirements for data availability concerning third-party data usage. In response to the guidelines provided, we have added a section to our manuscript titled "Data Availability Statement". This section details the 4 points raised. As cited below:

*“Description of the data set and the third-party source: The dataset includes fMRI scans along with demographic information such as age and gender, and clinical data related to Alzheimer’s Disease status (normal control, mild cognitive impairment, or Alzheimer’s Disease). All data were obtained from the Alzheimer’s Disease Neuroimaging Initiative (ADNI).*

*Verification of permission to use the data set: The use of ADNI data is governed by the ADNI Data Use Agreement, which all researchers must accept before accessing the data. Our study complied with all terms of this agreement.*

*Confirmation of whether the authors received any special privileges in accessing the data that other researchers would not have: No special privileges were granted to the authors in accessing the ADNI data. All data used in this study are available to other researchers under the same terms through the ADNI database.*

*All necessary contact information others would need to apply to gain access to the data: Researchers interested in accessing ADNI data should visit the ADNI website at https://adni.loni.usc.edu/data-samples/adni-data/#AccessData. The website provides detailed instructions on the registration and data access application process.”*

We hope this addition adequately addresses the data availability requirements but are ready to modify should there be additional requests.

**3. Please include your full ethics statement in the ‘Methods’ section of your manuscript file. In your statement, please include the full name of the IRB or ethics committee who approved or waived your study, as well as whether or not you obtained informed written or verbal consent. If consent was waived for your study, please include this information in your statement as well.**

As requested, we have added the ethics statement in the 'Methods' section of our manuscript, as cited below:

*"This study involves secondary analysis of existing public data obtained from the Alzheimer's Disease Neuroimaging Initiative (ADNI). The original collection of the data was approved by the institutional review boards of all participating institutions. Informed consent was obtained from all participants involved in the study. This secondary analysis did not involve direct interaction with human participants, and therefore no additional ethical approval was required for this study."*

We want to stress that our study is a secondary analysis of publicly available data, and as such, does not necessitate additional ethical approval. We hope this clarification addresses the concerns and assists in the review process. We appreciate the guidance and the opportunity to clarify this aspect of our research.

**4. We note you have included a table to which you do not refer in the text of your manuscript. Please ensure that you refer to Table 3 in your text; if accepted, production will need this reference to link the reader to the Table.**

We appreciate the editor’s attention to detail regarding the reference to Table 3. We have included a reference to this table in the supplementary materials section to ensure that it is appropriately linked and accessible. We hope this update meets the journal's requirements. Please let us know if further adjustments are needed.

### Reviewers' comments:

**Reviewer #1: This manuscript describes a new machine learning algorithm using convolutional networks to detect Alzheimer’s disease from fMRI scan data. The manuscript is well written and the following minor comments and suggestions should be addressed:
1. The authors should consider re-organizing their figure placement such that it appears closer to the section where they are discussed.**

We sincerely thank the reviewer for the positive feedback on the clarity of our manuscript and the suggestion regarding the placement of figures. We would like to clarify that the separation of figures and text was due to the submission format guidelines. However, we fully acknowledge the value of the suggestion, we will work with the journal and do our best to reorganize the figures to appear closer to the sections where they are discussed.

**2. In the "Deep Learning Frameworks" section, more background information should be given on the baseline RNN and MLP models. The number of trainable parameters in all models should be listed for comparison.**

We thank the reviewer for commenting on the need for additional information. As a response, we have updated the section correspondingly in the new manuscript. For the convenience, we also place it as below:

*“Baseline Models*

*To provide a robust comparison, we utilized several baseline models, each designed to address different aspects of the data characteristics:*

- *RNN Model: The baseline Recurrent Neural Network (RNN) model is tailored to capture temporal dependencies within the fMRI data sequences. It utilizes gated recurrent units (GRUs) to address the vanishing gradient problem, making it suitable for learning from time-series data.*
- *MLP Model: The baseline Multilayer Perceptron (MLP) using a standard fully connected network architecture to process static features extracted from fMRI data.*

*Trainable Parameters*

*Here, we provide details on the trainable parameters for each model, demonstrating the computational efficiency of our proposed approach:*

- *our Model: Approximately 1.3 million (1,331,402) parameters.*
- *Gupta’s Model: Approximately 2.9 million (2,948,866) parameters.*
- *RNN Model: Approximately 12.6 million (12,595,586) parameters.*
- *MLP Model: Approximately 25.2 million (25,174,338) parameters.”*

**3. Some statistical analysis should be added to see if the performance improvement is statistically significant compared to Gupta's model.**

We thank the reviewer for emphasizing the importance of statistically validating the performance improvements of our model compared to Gupta's model. In response, we performed detailed statistical analyses, incorporating both parametric (T-test) and non-parametric (Wilcoxon signed-rank test) methods to assess the significance of the differences observed between the models across multiple datasets.

To provide a thorough evaluation, we analyzed the models' performances on the Resting and PW datasets. The statistical tests revealed that, while the T-test for the Resting dataset suggested only a trend towards significance (T-stat = -1.96, p = 0.0545), the Wilcoxon test indicated a significant difference (W-stat = 311, p = 8.73 * 10^-6^), indicating a consistent improvement in our model's performance. For the PW dataset, both the T-test (T-stat = -3.07, p = 0.0025) and the Wilcoxon test (W-stat = 6649, p = 2.31 * 10^-4^) demonstrated statistically significant improvements in model performance. These findings have been documented and discussed in the revised manuscript. We hope that these additions address the concerns raised and strengthen the manuscript. For reviewer’s convenience, we cited the related parts as below:

*“We also performed statistical analyses of our and Gupta's model performances on different datasets, as summarized in Table \ref{tab:model_comparison}. For the Resting test dataset, the T-test (T-stat = -1.96, p = 0.0545) suggested a trend towards a difference in model performance that did not reach conventional statistical significance. However, the significant result from the Wilcoxon test (W-stat = 311, p = $8.73 \times 10^{-6}$) indicates a consistent difference in the median of the ranked differences, suggesting that one model generally outperformed the other. For the PW dataset, both the T-test (T-stat = -3.07, p = 0.0025) and the Wilcoxon test (W-stat = 6649, p = $2.31 \times 10^{-4}$) showed significant differences in model performance, indicating consistent discrepancies between the models across this dataset. The significant values from both the T-test and Wilcoxon test for the PW dataset confirm robustness in performance, suggesting that our model is more suited to external conditions represented by this dataset, while performing as well as the Gupta's model.”*

**4. Did the authors use any data augmentation techniques to compensate the issue of class imbalance? Why or why not? The authors should provide some discussion on this.**

We thank the reviewer for bringing up the important issue of class imbalance and the use of data augmentation techniques to address it. In our study, we did not use data augmentation techniques to handle class imbalance, primarily due to concerns about potentially compromising the authenticity and clinical relevance of the complex fMRI data. We were cautious about introducing synthetic variations that might not accurately reflect the underlying biological and medical realities, which are important in the study of Alzheimer’s Disease using fMRI. However, recognizing the importance of this issue, we have added discussion in the "Areas for Improvement" section of our manuscript, as cited below:

*“Moreover, addressing class imbalance remains a critical challenge in the effective training of predictive models. In this study, we opted not to use data augmentation techniques to mitigate class imbalance, due to concerns about the authenticity and representation of the complex fMRI data. This decision, while aligned with our goal to preserve data integrity, suggests an area for improvement. Future research could explore the development and validation of sophisticated data augmentation techniques that are capable of enhancing dataset balance without compromising the quality and reliability of the data. This exploration is crucial for improving model performance and ensuring its clinical viability.”*

**5. Figure 3. The authors should indicate which model was used to generate this plot. If the authors' model was used, it might be worthwhile to compare that with a similar plot generated from Gupta's model for comparison.**

We thank the reviewer for the suggestion to clarify which model was used to generate the plots in Figure 3. In response, we have clarified that the plots were generated from our model, while also included plots generated using Gupta's model. To maintain the focus of the main text on our proposed model, we have placed these comparative plots in the supplementary materials. We believe this approach keeps the manuscript streamlined while still providing the detailed comparative data for interested readers. However, we acknowledge the importance of direct comparison and are prepared to incorporate these figures into the main manuscript if the reviewer or editorial board deems it essential for understanding the full context of our study. We are open to making any necessary adjustments to meet the journal's standards and enhance the article's comprehensiveness.

*“For further comparative analysis between our proposed model and Gupta's model, we have included additional plots in the supplementary materials.”*

**Reviewer #2: - ## paper summary:
- This study aims to demonstrate feasibility of using fMRI + standard CNNs to predict risk of AD diagnosis with high performance despite low sample size and highly imbalanced training data. Post-hoc CNN feature map/SHAP explanations suggest that authors' CNN model relies on clinically known brain regions for assessing AD risk.
- ## strengths:
1) publicly available code
2) post-hoc model interpretation seems to align with domain knowledge of AD/MCI
- ## major issues:
1) the scientific goal, focus, or overall argument/hypothesis/structure of the study is not evident
- Q: why is the focus of the study to build a CNN? what purpose is it expected to serve given prior models with AUROCs/accuracies above 0.95/95%?
- the introduction is not thematically aligned with the running title of the paper**

We thank the reviewer for the careful analysis and valuable comments, which have provided us with an opportunity to better articulate the aims and significance of our study. Below, we address the points raised:

Our study initially aimed to explore the feasibility of a multimodal approach that incorporates both genetic and fMRI data for predicting Alzheimer's Disease (AD). However, early results indicated that the addition of genetic data did not significantly enhance the predictive performance given the constraints of our dataset—small size and the imbalanced samples. This thus prompted a more focused investigation into an imaging-only model using Convolutional Neural Networks. We optimized the CNN based on this finding and demonstrated it to be more suited for handling the complexities of the data. To further enhance the utility and transparency of the neural network, we integrated interpretability features using techniques such as SHapley Additive exPlanations (SHAP) values, providing insights into potential areas that have higher influence in predicting AD, thereby increasing the clinical relevance and trustworthiness of our predictions.

We chose CNNs due to their superiority in feature extraction from raw imaging data, as well as its less demanding of computing resources, all are points worth considering when dealing with high-dimensional spaces with potentially insufficient computing resources in fMRI studies. CNNs are advantageous for their ability to automate the feature extraction process, reducing the need for extensive preprocessing and making the model more adaptable and easier to implement in clinical settings. This less trainable parameters approach also allows for better scalability and adaptability to various data-rich settings, potentially improving model performance as more data become available. In addition, the feature maps of CNN in the first layer adds a layer of interpretability on medical images, compared to other models such as RNNs. We appreciate the opportunity to clarify that, according to our literature review, no prior models have demonstrated a higher AUROC consistently than our CNN on the datasets we used. While models like Gupta's have shown high specificity on specific datasets, it did not generalize well when applied to our imbalanced and small sample size datasets. Additionally, the limited availability of code from recent publications, or the significant adjustments required to apply them to our case, constrained our comparisons.

We appreciate the reviewer’s observation regarding the alignment of our introduction with the paper’s title. We have carefully revised the introduction to better reflect the focus on convolutional neural networks and their application to fMRI data for Alzheimer’s disease prediction, as indicated in our title. Given the comprehensive nature of these revisions, we invite the reviewer to examine the updated introduction in the revised manuscript to assess the enhancements.

**2) lack of overall scientific contributions:
- Q: what exactly is the knowledge gap in past literature that this study wants to address/explore or is motivated by?
- related literature and its limitations must be summarized under above context and not simply enumerated.
- Q: is the only issue that past models/code were not publicly released?
- Q: what novel insight is the reader supposed to take away from the interpretability analysis?**

We appreciate the comments regarding the scientific contributions of our study. Below, we address each of your questions to clarify these aspects:

Knowledge Gap: The primary knowledge gap our study addresses is the application of deep learning techniques, specifically 3D Convolutional Neural Networks (CNNs), to functional magnetic resonance imaging (fMRI) data for Alzheimer’s disease (AD) prediction. While previous studies have utilized CNNs for AD detection, our research extends this by emphasizing an interpretable deep learning framework that enhances understanding of how model decisions are made, which is often underexplored in existing literature.

Limitations of Related Literature: Previous studies in this domain have often focused on model performance metrics such as accuracy and AUC but have provided limited insights into the "why" behind model predictions. Besides the lack of readily available models, which hinders reproducibility and progress speed across studies, many of these models also suffer from the "black box" syndrome, where decision-making processes are opaque. As for the novel insights from interpretability, the interpretability aspect of our study is designed to provide novel insights into which specific features within the fMRI data are most indicative of AD, according to the CNN. By utilizing SHAP values, we offer a detailed exploration of feature importance across brain regions, which can aid clinicians and researchers in understanding potential biomarkers for early AD detection. This approach can help bridge the gap between high performing predictive models and clinical appreciation. We have updated the manuscript to discuss these aspects, as cited below:

*“The primary knowledge gap our study addresses is the novel application of 3D CNNs to fMRI data for AD prediction. Unlike previous studies that primarily focused on detection accuracy, our research extends these efforts by introducing interpretable aspects. This not only enhances the understanding of model decisions but also addresses the often underexplored aspect of explainability.”*

**3) lack of clinical contribution:
- study maybe based on a flawed/irrelevant experimental design - not sure if CN vs AD is a clinically relevant classification task at all (although it is convenient for training a ML/DL classifier). Given that this model and others before it all have achieved very high AUC/accuracy, why are these models not yet trusted for use in real-world clinical workflows? Perhaps a design that focuses on CN vs MCI may be more clinically impactful?
- the interpretability analysis seems highly speculative/cherry-picked and affected by authors' confirmation bias, no expert-in-the-loop clinical assessment was done on the SHAP explanations/CNN feature maps nor were any counter-intuitive associations found/reported. The (presumably) overlapping train/val/test data splits during training further complicates the reliability of any post-hoc model interpretation.**

We appreciate the comments regarding the clinical relevance of our classification tasks and the interpretability of our model. We understand these concerns and provide the following clarifications and justifications for our study design and methods:

- Relevance of CN vs. AD Classification: The primary focus on distinguishing between CN and AD subjects in our study was driven by the objective to enhance early detection mechanisms for AD using neuroimaging data. While we acknowledge that incorporating MCI as a distinct class could offer more granular insights into the progression stages of AD, our current dataset and model aimed to first establish a robust framework for the more binary distinction relevant in initial screenings. The limitations posed by the dataset size and balance issues further constrained the feasibility of including MCI without risking the model's ability to learn effectively, as evidenced by our report's statistical trends. However, the suggestion is well-taken, and we agree that expanding the classification to include MCI could substantially increase the clinical applicability of our model. We plan to extend our model to expand the dataset to include MCI in future studies, using larger datasets that will allow such a tri-class classification to be more effectively implemented and validated.
- Interpretability and Expert Involvement: We acknowledge that the interpretability analysis, primarily based on SHAP values, might appear speculative without direct clinical validation. To enhance the reliability of our interpretations, we have included detailed analyses and additional results (plots) in the supplementary documents. We recognize the importance of involving clinical experts in neurodegenerative diseases to validate and refine our model's interpretability. However, due to current limitations in budget and time, we are unable to establish these collaborations immediately. Despite these challenges, we are committed to integrating 'expert-in-the-loop' systems to help in identifying and mitigating biases and ensuring that the model's focus aligns with clinically relevant features.
- Data Integrity and Experiment Design: Regarding the data splits and the potential for overlap, we ensured strict partitioning of data in our experiments to avoid any leakage. Each data was exclusively assigned to one of the sets (training, validation, or testing). Details of these methods can be found in our open GitHub repository for reproducibility and integrity.

We have updated the areas for improvements section correspondingly, as cited below:

*“The primary focus on distinguishing between CN and AD subjects in our study was driven by the objective to enhance early detection mechanisms for AD using neuroimaging data. While we recognize that incorporating MCI as a distinct class could offer more granular insights into the progression stages of AD, our current dataset and model aimed to first establish a robust framework for the more binary distinction relevant in initial screenings. The limitations posed by the dataset size and balance issues further constrained the feasibility of including MCI without risking the model's ability to learn effectively. However, expanding the classification to include MCI could substantially increase the clinical applicability of our model. The potential to improve clinical applicability can be expanded to include Mild Cognitive Impairment (MCI) using a larger dataset for more granular insights and a tri-class classification system. This advancement would not only improve the model's sensitivity to AD-specific neuroimaging biomarkers but would also incorporate domain-specific knowledge to significantly enhance the predictive accuracy and clinical applicability of the findings.*

*On the other hand, the interpretability analysis may be perceived as speculative without direct clinical validation. Recognizing the importance of involving clinical experts in neurodegenerative diseases, the integration of 'expert-in-the-loop' systems could be beneficial for future research as resources allow. This enables identifying and mitigating biases and ensuring that the model's focus aligns with clinically relevant features, thereby potentially enhancing the predictive accuracy and clinical applicability of the findings.”*

**4) experiment design: Q: what is the point of comparing your model with Gupta's study? What makes Gupta's study relevant to yours? In general, what is the purpose of the Table 2 comparisons and how do they support the goal of this study?**

Thank you for your questions regarding our experiment design and the specific comparisons made within our study. We hope following answers address the points raised by the reviewer:

Relevance of Gupta's Study: Gupta’s study is a recently published study in the field, with code readily available for use; their study performs AD prediction using deep learning models, which involves training CNNs originally designed for 2D images. Their methodology closely aligns with our objective of enhancing AD prediction models. By comparing our model to Gupta’s, we aim to demonstrate improvements in performance and efficacy that can be achieved through our architectural design.

Purpose of Table 2 comparisons: By comparing our model to Gupta’s and baseline models (RNN and MLP), we provide a benchmark to evaluate the relative improvements our approach offers in terms of accuracy, AUC, and other metrics, demonstrating the advantages of our models over existing methods and classical methods. Our design choices, such as the use of different layer structures or activation functions, are underscored by comparing performance metrics. Showing how these choices impact model performance help validate our architectural and procedural decisions. The comparisons fundamentally support the goal of our study by demonstrating that our model can handle the intricacies of AD prediction effectively, particularly in dealing with complex, multimodal datasets, while also showing that our model can achieve better performance on tasks that are similar to real-world clinical settings, supporting its potential utility. It also highlights the specific strengths of our approach in handling data imbalances, which are preferable for adoption in medical diagnostics.

**5) results: (Table 2) Judging by the 0.0 F1 and 0.50 AUC scores, none of these models seem to be trained properly. Are all high AUCs simply due to severe class imbalance in the test set? If so, what was done to protect against/reduce the impact of class imbalance during model training?**

We appreciate the reviewer's comment on the class imbalance and data augmentation techniques in our study. We didn’t employ data augmentation for balancing the classes due to the complex and delicate nature of fMRI data in AD research. Our primary concern was to preserve the authenticity and clinical relevance of the data. Introducing synthetic variations might distort the underlying biological and medical realities essential for accurate diagnosis and understanding of AD. In addition, despite these constraints, our model's ability to perform better without data augmentation highlights its robustness and adaptability to real-world datasets. This approach ensures that our findings are based on realistic data characteristics and the model is suited for clinical application. We acknowledge the potential benefits of addressing class imbalance more explicitly and appreciate the constructive suggestion to explore this further. As such, we have included a discussion on potential strategies for handling class imbalance in the "Areas for Improvement" section of our manuscript, as cited below:

*“Moreover, addressing class imbalance remains a critical challenge in the effective training of predictive models. In this study, we opted not to use data augmentation techniques to mitigate class imbalance, due to concerns about the authenticity and representation of the complex fMRI data. This decision, while aligned with our goal to preserve data integrity, suggests an area for improvement. Future research could explore the development and validation of sophisticated data augmentation techniques that are capable of enhancing dataset balance without compromising the quality and reliability of the data. This exploration is crucial for improving model performance and ensuring its clinical viability.”*

**6) evaluation scheme:
- (line 118) Q: were the train/val/test data splits done with overlapping subjects? in other words, did the fMRI data slices of one subject end up in both train and test sets?
- Q: Are the classification results reported on test data collected from a seen site (whose data is present in train or val set) or previously unseen ADNI site? Overall, a rigorous evaluation scheme should test the model on an unseen ADNI site, using data of unseen subjects.**

Thank you for your questions regarding our evaluation scheme. We ensured non-overlap of data between the training, validation, and testing datasets to maintain the integrity of our evaluation process. Concerning the utilization of completely unseen data, particularly from new sites, we encountered specific challenges with the Perfusion Weighted (PW) dataset due to mismatches in data dimensions, which precluded its use as an external validation set. This limitation highlights the need for incorporating broader datasets in future studies to enhance model validation. As future and ongoing work, we are actively seeking additional datasets that align with our model’s requirements to address this issue, aiming to test our model’s performance on entirely new and unseen data from diverse sources.

**7) lack of methodological novelty or technical contributions: all the presented insights on class imbalance and low sample size are well-known in the ML/DL for healthcare community, this study does not present any new approach that can address any of the known/acknowledged issues.**

We appreciate the reviewer's comments regarding the methodological novelty and the presentation of known challenges such as class imbalance and small sample size within our study. While it is true that these issues are well-recognized within the machine learning community, our primary contribution in this manuscript is the application of a streamlined 3D Convolutional Neural Network that demonstrates robust performance on these challenges in AD prediction using fMRI data. Our study aims not at introducing novel methods for handling class imbalance or small sample size, but rather a carefully optimized CNN, when facing these obstacles, can achieve considerable accuracy and robustness in different datasets. For instance, despite the small size of our dataset, our model's architecture is able to perform better than other published and common methods. This indicates the model's ability to capture features in limited data scenarios. We also have choices in the model architecture and training process to mitigate the effects of class imbalance. For instance, our use of dropout layers is to reduce overfitting in imbalanced datasets. The effectiveness of these designs is shown in the consistent performance across the external PW dataset, suggesting the model’s capability to generalize despite the imbalance. Additionally, our optimized model achieved simplicity with significantly fewer trainable parameters, which are updated in the manuscript (and cited below for reference). This simplicity requires fewer resources and provides better interpretability, aligning with the needs of clinical applications where computational efficiency and model transparency.

*“Trainable Parameters*

*Here, we provide details on the trainable parameters for each model, demonstrating the computational efficiency of our proposed approach:*

- *our Model: Approximately 1.3 million (1,331,402) parameters.*
- *Gupta’s Model: Approximately 2.9 million (2,948,866) parameters.*
- *RNN Model: Approximately 12.6 million (12,595,586) parameters.*
- *MLP Model: Approximately 25.2 million (25,174,338) parameters.*

While these contributions may not as significantly extend the boundaries of new algorithmic developments, they provide considerable value to the field by applying existing methodologies effectively to solve real-world problems of clinical studies.*“*

**- ## minor issues:
1) manuscript needs significant language and scientific prose/style/structure review:
- one example: ROC plot/performance metrics and interpretability tools must first be introduced in methods section, not in results section!**

We thank the reviewer for the comments regarding the organization of our manuscript, specifically the placement of information about the ROC plots, performance metrics, and interpretability tools. In response, we revised the manuscript to introduce the ROC plots and performance metrics within the Methods section, as cited below:

*“Evaluation Metrics and Tools*

*To ensure a comprehensive assessment of our model's performance, we employed several evaluation metrics and tools, which are detailed below:*

*Receiver Operating Characteristic (ROC) Curves*

*The Receiver Operating Characteristic (ROC) curve is a graphical plot that illustrates the diagnostic ability of a binary classifier system as its discrimination threshold is varied. It is used to plot the True Positive Rate (TPR, sensitivity) against the False Positive Rate (FPR, 1-specificity) for different threshold settings. The Area Under the ROC Curve (AUC) provides a single measure of overall model performance across all classification thresholds, with a value of 1 representing perfect accuracy and 0.5 denoting a result no better than chance.*

*Performance Metrics*

*The following metrics were calculated to evaluate the performance of our deep learning models:*

- *Accuracy: The proportion of true results (both true positives and true negatives) among the total number of cases examined.*
- *Precision: The ratio of correctly predicted positive observations to the total predicted positives. It is a measure of the accuracy provided that a specific class has been predicted.*
- *Recall (Sensitivity): The ratio of correctly predicted positive observations to all observations in actual class.*
- *F1 Score: The weighted average of Precision and Recall. This score takes both false positives and false negatives into account.*
- *Matthews Correlation Coefficient (MCC): A coefficient that produces a high score only if the prediction obtained good results in all of the four confusion matrix categories (TP, FN, TN, FP), relative to the size of positive elements and negative elements in the dataset.*

*Interpretability Tools*

*SHAP (SHapley Additive exPlanations): SHAP values interpret the impact of having a certain value for a given feature in comparison to the prediction. This approach originated from game theory and provides a foundation for interpreting model output. In our study, SHAP values were utilized to highlight the most influential features in our model’s predictions, potentially identifying key brain regions in fMRI scans related to Alzheimer’s Disease.”*

We hope these changes address the concerns raised and improve the manuscript’s readability.

**2) (line 126) missing technical signal preprocessing details**

We thank the reviewer for their attention to the details of our signal preprocessing methods. While we considered these preprocessing steps to be foundational and straightforward for researchers familiar with fMRI data analysis, we appreciate the need for clarity to ensure the reproducibility of our results. To address this, we have added a sentence in the manuscript that directs readers to our GitHub repository where the complete preprocessing code is available (as cited below). We hope this amendment satisfactorily addresses the reviewer’s concerns and enhances the manuscript's utility to the research community.

*“The details and code for preprocessing are available in our GitHub repository (\url{https://github.com/gersteinlab/fmgene/tree/master}).”*

**3) should remove all gene-related details from the main text if focus is entirely on fMRI analysis**

We appreciate the reviewer's suggestion regarding the exclusion of gene-related details from the main text. While our initial research approach included both genetic and fMRI data to develop a comprehensive predictive model, we focused on the imaging-only model after finding that genetic factors did not significantly enhance AD prediction capabilities. The inclusion of gene-related details serves to provide context on the scope of our initial study objectives and the challenges encountered, which are used for a complete understanding of our research process. However, if the reviewer feels strongly about this aspect, we are open to amending the text to focus more directly on the fMRI analysis. We value your feedback and are willing to make necessary adjustments to align with the journal's focus and readers' expectations.

**4) (line 169) can move all NN related details to supplement since these are all standard and well-known.**

We appreciate the reviewer’s suggestion to move the network details to the supplementary materials to streamline the main text. In line with this suggestion, we have relocated the "Network Parameters" section, originally detailed from line 169 to line 196, to the supplement as technical details. We inserted a note in the main text where these details were originally discussed, as cited below:

*“Details on the specific architecture and parameters of our CNN model are provided in the Supplementary Information.”*

We hope this change will improve the readability of our manuscript without omitting any critical information.

**5) (line 197) Q: what exactly makes the CNN architecture "unified" and not simply a standard CNN? How exactly is adaptation to different input sizes achieved with a fully connected layer (line 183)?**

Thank you for your questions concerning the architecture of our CNN model. The term "unified" in our manuscript refers to the model's ability to process different types of fMRI data (Resting and Perfusion Weighted) consistently using the same architecture. This is achieved by designing the convolutional layers to handle the volumetric nature of fMRI data effectively, ensuring that the feature extraction process remains consistent. This approach allows for the simultaneous analysis of different fMRI datasets within the same framework, thus maintaining uniformity in feature extraction and classification methodologies. The adaptation to different input sizes is managed through the design of the fully connected layers. Specifically, the input features to the first fully connected layer vary depending on the dataset, allowing the network to adapt to different data sizes and complexities. This flexible input feature size helps accommodate variations in the input data without requiring changes to the overall architecture. We hope this response adequately addresses your questions and clarifies the design choices of our CNN architecture.

**6) (table 1) Q: Are the healthy controls age-matched and sex-matched to the AD/MCI patients? If not, the model may have learnt to predict age or sex instead of disease state.**

Thank you for the insightful comment regarding the age and sex matching of the healthy controls to the AD/MCI patients. In our current analysis, we did not explicitly match the healthy controls with the AD/MCI patients in terms of age and sex, primarily because in our binary classification task, we assigned the MCI group to class 0, which left only a limited number of AD cases for prediction. We recognize that this approach could lead to potential confounding effects, as differences in age or sex distributions across groups might impact the model's learning. Acknowledging the importance of this issue, we propose to address it in future revisions of our study. One approach we are considering is to expand the dataset to allow for more stringent matching criteria. Additionally, we plan to include these demographic variables as covariates in our model to help mitigate any potential bias. We hope this adjustment will ensure that the model predicts disease status based on relevant pathological features rather than demographic differences.

**7) Q: how was data normalization done for this multi-centre dataset, assuming site-specific effects exist and are non-negligible? is this normalization/standardization process used/documented previously by the ML/DL for fMRI community?**

In our study, we implemented standard z-scoring to ensure uniformity across the multi-center fMRI dataset. Additionally, to refine our model's input handling and enhance its training stability, we employed batch normalization within our neural network architecture. This method normalizes the input layer by adjusting and scaling the activations, helping to mitigate internal covariate shift caused by the diverse data characteristics inherent to multi-site datasets. Both normalization techniques are widely used by the community, ensuring that our methods align with common practices in the field.

**- ## misc. questions/comments on interpretability:
1) maybe an interesting ablative experiment: if certain brain regions are known to be associated with AD, does training models after masking those brain regions steeply hurt the model performance or does it learn to use some other feature of the data instead?**

We appreciate this suggestion the interesting suggestion regarding the ablative experiment. In our current study, we primarily focused on using unaltered fMRI scans to detect AD. The idea of masking known AD-related brain regions to examine how the model adapts and whether it finds alternative features for classification is an innovative approach to test the robustness and flexibility of our model. However, it is difficult to identify and mask off the regions accurately - for instance, we know the hippocampus tends to be a related region, but different patients have different scans properties, such as the size and location, which makes it a considerable challenge. On the other hand, the suggestion aligns well with potential research directions. For instance, we may pool the most focused important areas highlighted by SHAP, mask these areas and see how the model performs.

**2) robustness of interpretation: most model explanation methods have known limitations and robustness issues, i.e., their outputs are not always reliable. There is a serious general issue of author bias when looking at model interpretability results - we overlook the results that we don't expect to see and focus more on what matches our expectations. Some additional experiments are needed to establish robustness of presented insights.**

We acknowledge the reviewer's comment regarding the robustness of model interpretation methods. As highlighted in our study, interpretability tools such as SHAP values are utilized to identify influential brain regions in AD prediction using fMRI data. These tools provide valuable suggestions into the decision-making process of our model, particularly in distinguishing AD patients from controls. While our findings align with established neuroscientific knowledge, we recognize the inherent limitations and potential biases in interpretability analyses. Specifically, there is a risk of confirmation bias, where interpretations may inadvertently favor expected outcomes. To address concerns about the speculative nature of our interpretability analysis, as discussed earlier, we have included additional SHAP plots and feature maps in the supplementary documents. We hope this can alleviate the reviewer’s concern on robustness.

**3) model convergence: Q: Do all the compared models use the same set of brain regions when assessing AD risk? or do you get different explanations for each model?**

Yes, all the compared models utilize the same set of brain regions when assessing AD risk as they are all trained using the same dataset of 3D fMRI scans from the ADNI. It's important to note that while the baseline RNN and MLP models require preprocessing steps such as flattening the 3D scans into lower dimensions to fit their input requirements, they do receive the same information as the CNNs: the fundamental input remains consistent across all models. This ensures that each model is evaluating the same anatomical features.

**Reviewer #3: The paper proposed an improvement for Alzheimer's Disease (AD) early detection by utilizing 3D Convolutional Neural Network (CNN) to analize fMRI scans. Deep learning has proven effective in many domains, and its application to AD diagnosis represents a novel and powerful approach. The authors employed a substantial dataset and conducted rigorous experiments, demonstrating that 3D CNN-based analysis of fMRI scans achieves high accuracy. Furthermore, their results indicate that gene processing—a commonly used method for AD detection—is less effective than the proposed 3D CNN approach. For future development, I recommend that the authors design a neural network architecture specifically tailored for AD fMRI analysis.**

We sincerely thank the reviewer for the positive and insightful feedback on our study and for recognizing the potential of 3D Convolutional Neural Networks (CNN) in the early detection of Alzheimer's Disease (AD) using fMRI scans. We are encouraged by your acknowledgment of our findings. For future development, we have modified the manuscript according to the comments based on your comments, as well as re-organized the structure, where the most relevant part is cited below:

*“The primary focus on distinguishing between CN and AD subjects in our study was driven by the objective to enhance early detection mechanisms for AD using neuroimaging data. While we recognize that incorporating Mild Cognitive Impairment (MCI) as a distinct class could offer more granular insights into the progression stages of AD, our current dataset and model aimed to first establish a robust framework for the more binary distinction relevant in initial screenings. The limitations posed by the dataset size and balance issues further constrained the feasibility of including MCI without risking the model's ability to learn effectively. However, expanding the classification to include MCI could substantially increase the clinical applicability of our model. The potential to improve clinical applicability can be expanded to include MCI using a larger dataset for more granular insights and a tri-class classification system. This advancement would not only improve the model's sensitivity to AD-specific neuroimaging biomarkers but would also incorporate domain-specific knowledge to significantly enhance the predictive accuracy and clinical applicability of the findings.*

*…*

*Lastly, the exploration of advanced or novel algorithms holds the potential to enhance predictive performance further. Investigating methods to address imbalanced datasets, such as the application of synthetic data generation techniques like generative adversarial networks (GANs) or employing advanced sampling methods, could significantly refine model efficacy. Moreover, in furthering the efficacy of AD detection using fMRI scans, an architecture specifically optimized for AD-related neuroimaging data could refine the model's ability to detect nuanced patterns specific to AD”*
